# [Retracted] PYGB siRNA inhibits the cell proliferation of human osteosarcoma cell lines

**DOI:** 10.3892/mmr.2026.14014

**Published:** 2026-09-10

**Authors:** Shuwei Zhang, Yichi Zhou, Yuanyu Zha, Yang Yang, Linlong Wang, Jingfeng Li, Wei Jin

Mol Med Rep 18: 715–722, 2018; DOI: 10.3892/mmr.2018.9022

Following the publication of the above paper, it was drawn to the Editor's attention by a concerned reader that certain of the western blot data featured in Fig. 5A on p. 721 had already been published in an article in the journal *Experimental & Molecular Medicine* that was written by different authors at different research institutes, in addition to reappearing in other articles subsequently received and published in other journals that were also written by different authors at different research institutes. A subsequent investigation of this paper undertaken by the Editorial Office also revealed that flow cytometric data in Fig. 3A and B had already been published previously in other articles that were written by different authors at different research institutes, and the control GAPDH blots featured in Fig. 1B were strikingly similar to blots that were published in the years 2018–2020 in three other articles written by different authors.

Owing to the fact that the abovementioned data in this article had already been published prior to its submission to *Molecular Medicine Reports*, the Editor has decided that this paper should be retracted from the Journal. The authors were asked for an explanation to account for these concerns, but the Editorial Office did not receive a satisfactory reply. The Editor apologizes to the readership for any inconvenience caused.

